# Determinants of dropout from lifestyle interventions for overweight polycystic ovary syndrome: An exploratory analysis of randomized controlled trial

**DOI:** 10.1371/journal.pone.0351575

**Published:** 2026-06-26

**Authors:** Min Xie, Siyu Zhou, Jing Wu, Xiaoyan Luo, Yichuan Guo, Lin Qiao, Jing Zhang

**Affiliations:** 1 West China Second University Hospital, Department of Obstetrics and Gynaecology, Sichuan University, Chengdu, China; 2 Chengdu Qingbaijiang People’s Hospital, Department of Obstetrics and Gynaecology, Chengdu, China; 3 Key Laboratory of Birth Defects and Related Diseases of Women and Children, Sichuan University, Ministry of Education, Chengdu, China; 4 Reproductive Endocrinology and Regulation Laboratory, West China Second University Hospital, Sichuan University, Chengdu, China; 5 Amsterdam Public Health Research Institute, Department of Public and Occupational Health, Amsterdam UMC, Amsterdam, the Netherlands; 6 West China Second University Hospital, Follow up centre, Sichuan University, Chengdu, China; Zhejiang University College of Life Sciences, CHINA

## Abstract

**Objective:**

This exploratory analysis of a randomized controlled trial aimed to identify baseline predictors of dropout in overweight women with polycystic ovary syndrome (PCOS) participating in a lifestyle intervention program.

**Methods:**

An exploratory analysis was conducted using data from a randomized controlled trial involving overweight or insulin-resistant women with PCOS aged 18–45. All participants received cyclic progestin, metformin, and a structured lifestyle intervention. Dropout was defined as proactive withdrawal, missing two consecutive visits, or loss of contact for more than six months. Univariate and adjusted multivariable logistic regression models were used to identify factors associated with dropout.

**Results:**

Among the participants, 61.06% (n = 69) dropped out within one year. No significant differences were observed in baseline demographic, clinical, biochemical, psychological, or dietary characteristics between completers and dropouts. However, baseline physical activity level (PAL), objectively measured using an accelerometer, was identified as the strongest predictor of dropout. Each 0.3-unit increase in PAL was associated with a 29.6% reduction in the likelihood of dropout.

**Conclusion:**

Baseline PAL is strongly associated with the risk of dropout. Screening for PAL in women with PCOS is recommended, and those with lower PAL should receive personalized support in addition to lifestyle interventions to improve adherence and promote weight loss.

## Introduction

Polycystic ovary syndrome (PCOS) is a prevalent and heterogeneous endocrine disorder affects 8% to 13% of reproductive-aged women globally, characterised by a constellation of interrelated reproductive abnormalities. Diagnosis is base on the presence of irregular menstrual cycles and/or hyperandrogenism, alongside either ultrasound evidence of polycystic ovarian morphology or elevated anti-müllerian hormone levels [1]. Additionally, PCOS is associated with the development of metabolic syndrome in up to 33% of affected women [2]. These combined reproductive and metabolic disturbances elevate the risk of major health complications over the lifespan, such as anovulatory infertility and type 2 diabetes [3], resulting in significant health and economic consequences.

While various theories have been proposed to elucidate the pathogenesis of PCOS, obesity emerges as a key pathophysiological component. Approximately 50% to 80% of women with PCOS are concurrently obese [4]. Moreover, evidence from Mendelian randomization analyses suggests a causal relationship between body mass index (BMI) and PCOS [5–7]. Obesity contributes to PCOS development through multiple mechanisms. For instance, the severity of obesity correlates with the extent of selective insulin resistance (IR). IR, along with consequent hyperinsulinemia, activates excessive ovarian androgen production, thereby accelerating PCOS development [8]. Additionally, obesity-related inflammation may exert potential effects on ovarian physiology due to dysregulated adipokine secretion, further impacting insulin sensitivity [9]. Collectively, it is well established that obesity exacerbates PCOS manifestations, while weight loss ameliorates the features of PCOS.

According to the international evidence-based guideline, first-line treatments for women with PCOS are lifestyle interventions, comprising dietary modification, exercise, and behavioural strategies [1]. Emphasis on prevention of weight gain and regular weight monitoring is crucial in PCOS management. Notably, achieving a targeted weight reduction of 5% to 10% demonstrated benefits in improving both reproductive and metabolic function indicators in women with PCOS [10]. However, existing clinical trials investigating the impact of weight loss in women with PCOS predominantly feature short to medium follow-up durations ranging from 4 weeks to 6 months, involving small sample sizes of 12–100 participants [11]. Despite effort to enhance adherence through more frequent visits in the intervention group, the rigorous lifestyle interventions employed, such as strict calorie restriction, often prove physiologically unrealistic and unsustainable [10]. Consequently, even in short-term follow-ups, considerable dropout rates are reported, potentially exaggerating treatment effects [11]. Furthermore, several medium- to long-term trials with follow-ups of at least 6 months reported dropout rates of 31% to 63% over six months, with a significant proportion of participants quitting the trial within the first 8 weeks [12–14]. It becomes evident that a single lifestyle prescription is unlikely to provide effective long-term management solutions.

Subsequently, growing attention has been directed toward maintaining adherence among women with PCOS undergoing lifestyle interventions and reducing high dropout rates in weight management. Identifying baseline characteristics linked with dropout is crucial for recognising individuals who could benefit from such interventions or who require alternative supports to achieve weight loss goals. While lower quality of life (QoL) score, higher baseline weight, elevated androstenedione level, elevated free and total testosterone level, and increased depressions score at baseline have been linked to higher dropout rates in prior studies [12–14], few investigations have explored dietary preferences, physical activity level (PAL), and body composition as predictors in overweight women with PCOS. Notably, these factors are robust predictors of weight loss maintenance in the general population, as supported by a systematic review [15]. The same review highlighted inconclusive value of depression scores and QoL due to the limited study numbers, warranting further investigation [15].

This exploratory analysis, conducted within a randomized controlled trial, aims to identify potential baseline characteristics associated with dropout during the lifestyle interventions in overweight women with PCOS. Baseline factors encompass menstrual characteristics, PCOS features, biochemical parameters, psychological scores, dietary and physical activity questionnaires, and objectively measured body composition, and exercise capacity.

## Materials and methods

### Study design and setting

This exploratory analysis used data from a randomized controlled trial (RCT) conducted at the PCOS clinic within the Department of Reproductive Endocrinology, West China Second University Hospital, China. The primary aim of RCT was to compare the efficacy of a network platform-based lifestyle intervention combined with medication against standard care in improving reproductive function among women with PCOS.

### Ethics statement

The study was approved by the Medical Ethics Committee of West China Second University Hospital, Sichuan University (Approval No. 2020(060)) and registered with the Chinese Clinical Trial Registry (ChiCTR2000034263) on June 20, 2020. All procedures adhered to the Declaration of Helsinki. Written informed consent was obtained from all participants prior to enrolment.

### Participants

Eligible women were recruited between September 20, 2020, and December 30, 2022. Inclusion criteria were: age 18–45 years; PCOS diagnosis per Rotterdam 2003 criteria; BMI > 23.9 kg/m² or HOMA-defined insulin resistance; no pregnancy intention within the next year; smartphone proficiency; and willingness to adjust lifestyle. Exclusion criteria included: conditions contraindicating dietary protein or exercise (e.g., chronic kidney disease, heart failure, severe joint or gastrointestinal disease); psychological disorders; malignant tumors; recent weight-loss medication/surgery (within 3 months); use of drugs affecting hormone or glucolipid metabolism; allergy to dydrogesterone or metformin; participation in other trials within the past month; and abnormal cervical cytology (excluding inflammatory lesions). The sample size calculation was based on a notable difference in ovulation rate as the primary outcome measure of this RCT. A total of 114 participants (57 per group) were enrolled, a number lower than the planned 130 (65 per group, accounting for a 10% dropout) due to recruitment challenges. Randomization(1:1 ratio) using a computer-generated random table, performed by a research doctor not involved in the study.

### Baseline Characteristics Measurements

The baseline characteristics included the following domains:

- Gynaecological factors: Menarche age, menstrual patterns (duration and cycle length), dysmenorrhea, gravidity, and parity (self-report/medical records).
- Anthropometry and body composition: Systolic/diastolic blood pressure, BMI, Waist-to-hip ratio (WHR). The Mobility Evaluation System (MES) (software version Mes-01S20, MaiDaKang, Beijing, China) assessed body fat percentage (BF%), strength of the lower limbs (SLL), range of motion (ROM), and PAL.
- PCOS characteristics: Clinical signs (Ferriman–Gallwey score, acne, baldness, acanthosis nigricans) were evaluated by specialists. Biochemical assays included insulin-related parameters (fasting, 1-hour, and 2-hour postprandial insulin; HOMA-IR(calculated as FINS×FPG/22.5)), glucose levels (fasting, 1-hour, and 2-hour postprandial glucose), lipid profile (total cholesterol and triglycerides), and reproductive hormones (estradiol, LH/FSH ratio, free androgen index, androstenedione, DHEAS, and SHBG). Sex hormone measurements were taken on days 2–5 of the menstrual cycle or during amenorrhea, other blood tests were cycle-phase independent.
- Lifestyle characteristics: Baseline psychological distress was assessed using the Huaxi Emotional-Distress Index (HEI), a validated scale for assessing depression and anxiety among Chinese patients [16,17]. PCOS-specific quality of life was measured using the PCOS Health-Related Quality of Life Questionnaire (Chi-PCOSQ) [18]. Physical activity was evaluated using the long-form International Physical Activity Questionnaire (IPAQ) [19], and dietary habits using a revised version of the Semiquantitative Food Frequency Questionnaire (SQFFQ) [20]. All instruments have demonstrated satisfactory reliability and validity in previous studies.

### Interventions

All participants received standardized medication: cyclic dydrogesterone(20 mg/day for 14 days in the last half of the menstrual cycle) and metformin (1500 mg/day). In addition, they received structured lifestyle guidance, including dietary and physical activity guidance from a nutritionist. Participants were randomly allocated into one of two groups. The first group was invited to use a network interactive platform for self‐monitored and receiving monthly reminders; transmission was not mandatory. The second group received medication treatment and lifestyle modification without the platform or reminders.

### Outcomes

women with PCOS were advised to attend clinic every three months for weight assessment and prescriptions renewal. Dropout was defined as proactive withdrawal, missing two consecutive return visits, or loss of contact for over six months.

### Statistical analysis

Potential predictors were preselected based on the literature. Logistic regression was used to identify dropout-associated variables. A univariate analysis retained variables with p < 0.10. Multicollinearity among selected variables was examined using a correlation matrix; for highly correlated pairs (r > 0.80), the variable with greater clinical relevance and univariate significance was retained. A backward stepwise logistic regression was then performed, removing non-significant predictors (p > 0.05). The final model was assessed for goodness-of-fit using the Hosmer-Lemeshow test. Multicollinearity was further evaluated using tolerance and variance inflation factor (VIF). Model explanatory power was quantified by Nagelkerke's pseudo R², and discriminative ability by the area under the receiver operating characteristic (ROC) curve.

## Results

### Baseline characteristics of women with PCOS

**Fig 1:**
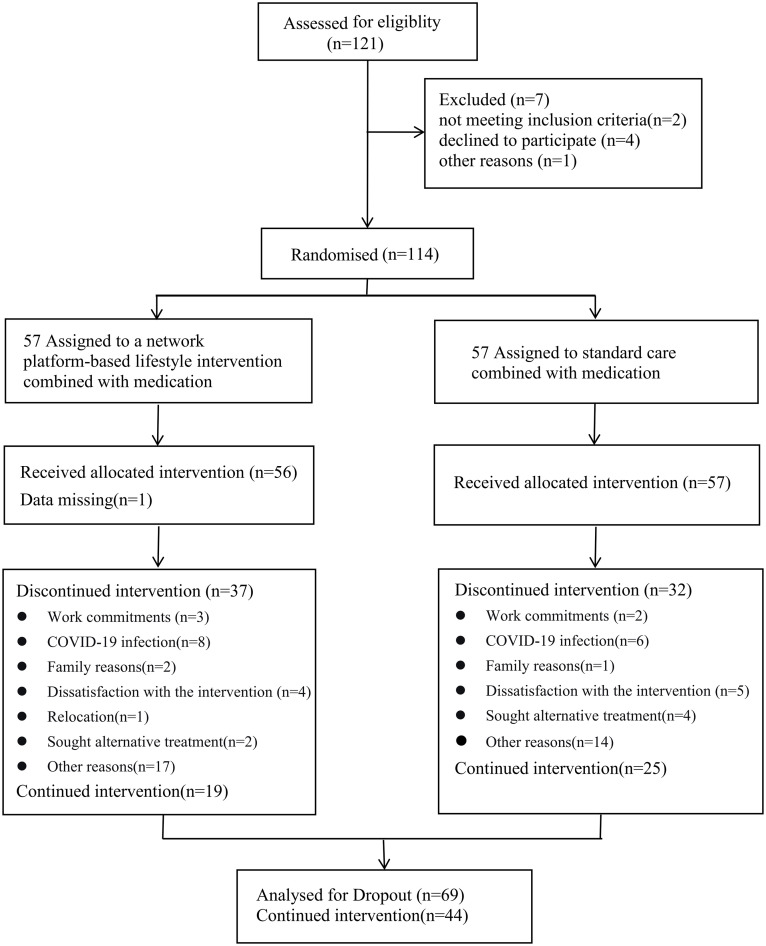
CONSORT flowchart.

A total of sixty-nine women (61.06%) dropped out of the lifestyle interventions (Fig 1), with no significant differences in attrition rates observed between the network interactive platform group and the control group (p = 0.444). Key baseline comparisons between dropouts and completers (Table 1) showed that dropouts had significantly lower PAL (1.42 vs. 1.50, p = 0.002) and higher BMI (27.99 vs. 26.35 kg/m², p = 0.028), as well as a higher prevalence of acanthosis nigricans (38.2% vs. 15.9%, p = 0.019). Trends toward higher gravidity, weight, and body fat percentage were observed in dropouts (p = 0.056–0.076). No significant differences were observed in psychological measures or most dietary factors. Except for lower vitamin C intake in the dropout group (p = 0.044), no other dietary differences were detected.

**Table 1:** Characteristics of study participants according to dropout status.

	Dropout (n = 69) Mean±SD/N(%)	None-dropout (n = 44) Mean±SD/N(%)	p-value
Intervention – no. (%)	37 (53.6)	20 (44.4)	0.444
Age – years	24.12 ± 4.01	24.48 ± 3.47	0.624
Gravidity history – no. (%)	12 (17.6)	2 (4.5)	0.076
Parity history – no. (%)	6 (8.7)	2 (4.5)	0.480
Menarche age – years	12.87 ± 1.22	12.64 ± 0.94	0.288
The longest period – days	11.72 ± 23.03	6.41 ± 1.02	0.130
The longest cycle – days	132.25 ± 111.47	130.30 ± 90.45	0.923
Dysmenorrhea – no. (%)	35 (51.5)	16 (36.4)	0.126
Systolic blood pressure – mmHg	119.54 ± 9.29	118.65 ± 11.91	0.682
Diastolic blood pressure – mmHg	78.78 ± 7.61	79.08 ± 9.05	0.861
Weight –kg	72.10 ± 11.57	67.80 ± 11.52	0.056
BMI – kg/m^2^	27.99 ± 3.99	26.35 ± 3.59	0.028*
WtHR	0.84 ± 0.05	0.84 ± 0.06	0.714
Ferriman Gallwey score	2.18 ± 3.36	1.91 ± 2.46	0.650
Mild acne – no. (%)	20 (29.4)	15 (34.1)	0.837
Moderate to severe acne – no. (%)	11 (16.2)	8 (18.2)	0.837
Baldness – no. (%)	14 (20.6)	9 (20.5)	1.000
Acanthosis nigricans – no. (%)	26 (38.2)	7 (15.9)	0.019*
HOMA-IR	5.59 ± 4.19	4.60 ± 2.28	0.153
Fasting insulin – µIU/ml	24.25 ± 17.00	19.75 ± 8.75	0.108
1 hour insulin - µIU/ml	171.02 ± 84.80	153.84 ± 73.93	0.412
2 hour insulin - µIU/ml	151.47 ± 89.69	157.22 ± 93.26	0.750
Fasting glucose –mmol/L	5.17 ± 0.59	5.10 ± 0.39	0.516
1 hour glucose – mmol/L	8.86 ± 2.26	8.82 ± 2.50	0.953
2 hour glucose – mmol/L	7.31 ± 1.98	7.78 ± 1.93	0.229
TC – mmol/L	4.53 ± 0.91	4.41 ± 0.89	0.537
TG – mmol/L	1.62 ± 0.83	1.62 ± 0.89	0.997
E2 – pg/mL	59.53 ± 27.46	63.43 ± 47.64	0.591
LH/FSH ratio	1.86 ± 0.80	1.94 ± 0.71	0.586
FAI	9.06 ± 4.83	7.84 ± 4.40	0.181
DHEAS – µg/dL	282.64 ± 109.14	278.25 ± 107.93	0.835
AND – ng/mL	4.25 ± 1.28	4.77 ± 1.67	0.061
SHBG – nmol/L	20.46 ± 11.83	23.14 ± 10.04	0.219
Huaxi emotion index	9.35 ± 7.43	7.37 ± 5.05	0.127
PCOSQ	57.25 ± 25.55	54.60 ± 18.44	0.558
SL	1.15 ± 0.21	1.16 ± 0.21	0.812
ROM	143.65 ± 9.32	144.00 ± 7.92	0.838
Body fat percentage	40.95 ± 7.31	38.50 ± 6.16	0.068
PAL	1.42 ± 0.12	1.50 ± 0.16	0.002*
Protein intake	85.04 ± 18.36	89.18 ± 17.34	0.235
Fat intake	84.81 ± 25.78	86.67 ± 21.10	0.689
Carbohydrate intake	237.66 ± 48.01	238.29 ± 46.54	0.945
Fiber intake	24.04 ± 9.01	26.59 ± 8.55	0.139
Vitamin C intake	193.66 ± 70.48	222.83 ± 79.84	0.044*
Folic intake	267.80 ± 103.71	288.40 ± 103.15	0.304
Protein intake percentage	16.54 ± 2.70	17.05 ± 2.72	0.333
Fat intake percentage	36.83 ± 8.20	37.17 ± 7.20	0.823
Carbohydrate intake percentage	46.63 ± 9.27	45.78 ± 8.27	0.623
Sedentary time – hours/week	53.89 ± 23.08	54.36 ± 21.83	0.914
Heavy physical activity time – hours/week	0.90 ± 1.84	1.97 ± 8.29	0.299
Moderate physical activity time – hours/week	1.97 ± 3.08	2.08 ± 3.15	0.847
Walk time – hours/week	5.90 ± 7.67	7.20 ± 6.73	0.361

BMI: body mass index, WtHR: Waist-to-hip ratio, HOMA-IR: Homeostatic Model Assessment for Insulin Resistance, TC: total cholesterol, TG: triacylglycerols, E2: estradiol, LH: luteinizing hormone, FSH: Luteinizing hormone, FAI: free androgen index, DHEAS: dehydroepiandrosterone sulfate, AND: Androstenedione, SHBG: sex hormone binding globulin, PCOSQ: polycystic ovary syndrome questionnaire, SL: strength of lower limb, ROM: range of motion (knee-joint), PAL: physical activity level. *: p-value < 0.05.

### Unadjusted univariable logistic regression and correlation between predictive factors

Univariate analysis identified BMI, body fat percentage, weight, and PAL as associated with dropout (p < 0.10). Due to strong collinearity among adiposity indicators (pairwise Pearson correlations: weight vs. BMI = 0.89, weight vs. body fat percentage = 0.92, BMI vs. body fat percentage = 0.94), only BMI, which showed the strongest univariate association and a more readily interpretable effect size, was retained for the multivariable analysis (Tables 2 and 3).

**Table 2:** Univariate (unadjusted) model of the determinants of dropout from lifestyle interventions.

	No. of patients in analysis	OR	95% CI	p-value
Intervention	113	1.445	0.680-3.074	0.339
Age	113	0.975	0.883-1.077	0.621
Gravity history				
no	98	referee	referee	referee
yes	14	4.500	0.956-21.191	0.057*
Parity history				
no	105	referee	referee	referee
yes	8	2.000	0.385-10.385	0.410
Menarche age	112	1.210	0.852-1.716	0.287
The longest period	111	1.184	0.958-1.462	0.117
The longest cycle	112	1.000	0.996-1.004	0.922
Dysmenorrhea				
no	61	referee	referee	referee
yes	51	1.856	0.854-4.036	0.119
Systolic blood pressure	96	1.009	0.969-1.050	0.679
Diastolic blood pressure	96	0.995	0.946-1.047	0.859
Weight	113	1.034	0.999-1.070	0.059*
BMI	113	1.122	1.010-1.246	0.031*
WtHR	112	4.012	0.003-6312.34	0.711
Ferriman Gallwey score	112	1.031	0.905-1.173	0.648
acne				
no	58	referee	referee	referee
mild	35	0.757	0.321-1.783	0.524
moderate to severe	19	0.780	0.271-2.245	0.646
Baldness				
no	89	referee	referee	referee
yes	23	1.008	0.394-2.579	0.986
Acanthosis nigricans				
no	79	referee	referee	referee
yes	33	3.272	1.273-8.413	0.014*
HOMA-IR	111	1.100	0.961-1.260	0.168
Fasting insulin	112	1.028	0.992-1.065	0.124
1 hour insulin	63	1.003	0.996-1.009	0.405
2 hour insulin	107	0.999	0.995-1.004	0.747
Fasting glucose	111	1.287	0.605-2.739	0.513
1 hour glucose	64	1.007	0.812-1.248	0.952
2 hour glucose	107	0.885	0.725-1.080	0.229
TC	102	1.155	0.734-1.819	0.533
TG	103	1.001	0.625-1.604	0.997
E2	108	0.997	0.987-1.008	0.590
LH/FSH ratio	108	0.868	0.524-1.437	0.582
FAI	110	1.062	0.971-1.162	0.185
DHEAS	112	1.000	0.997-1.004	0.833
AND	112	0.774	0.587-1.021	0.070*
SHBG	110	0.979	0.946-1.013	0.221
Huaxi emotion index	111	1.049	0.986-1.116	0.130
PCOSQ	110	1.005	0.988-1.022	0.554
SL	113	0.800	0.130-4.941	0.810
ROM	113	0.995	0.953-1.040	0.836
Body fat percentage	113	1.055	0.995-1.117	0.071*
PAL	113	0.014	0.001-0.273	0.005*
Protein intake	113	0.987	0.967-1.008	0.234
Fat intake	113	0.997	0.981-1.013	0.686
Carbohydrate intake	113	1.000	0.992-1.008	0.944
Fiber intake	113	0.968	0.927-1.011	0.143
Vitamin C intake	113	0.995	0.989-1.000	0.049*
Folic intake	113	0.998	0.994-1.002	0.304
Protein intake percentage	113	0.933	0.811-1.073	0.331
Fat intake percentage	113	0.994	0.947-1.044	0.821
Carbohydrate intake percentage	113	1.011	0.969-1.055	0.619
Sedentary time	112	0.999	0.982-1.016	0.913
Heavy physical activity time	113	0.958	0.870-1.054	0.381
Moderate physical activity time	113	0.988	0.875-1.116	0.845
Walk time	113	0.976	0.927-1.028	0.365

OR: odds ratio, BMI: body mass index, WtHR: Waist-to-hip ratio, HOMA-IR: Homeostatic Model Assessment for Insulin Resistance, TC: total cholesterol, TG: triacylglycerols, E2: estradiol, LH: luteinizing hormone, FSH: Luteinizing hormone, FAI: free androgen index, DHEAS: dehydroepiandrosterone sulfate, AND: Androstenedione, SHBG: sex hormone binding globulin, PCOSQ: polycystic ovary syndrome questionnaire, SL: strength of lower limb, ROM: range of motion (knee-joint), PAL: physical activity levels. *: p-value<0.10.

**Table 3:** Correlation matrix of the determinants derived from univariate models.

	gravidity history	acanthosis nigricans	weight	BMI	A4	body fat percentage	physical activity level	Vitamin C intake
gravidity history	1.00							
acanthosis nigricans	0.18	1.00						
weight	0.07	0.39	1.00					
BMI	0.07	0.49	0.89	1.00				
A4	−0.03	0.00	0.02	0.04	1.00			
body fat percentage	0.09	0.40	0.92	0.94	0.03	1.00		
PAL	−0.06	−0.07	−0.14	−0.12	0.01	−0.16	1.00	
Vit C intake	−0.05	0.07	0.26	0.23	0.05	0.21	0.55	1.00

BMI: body mass index, A4: Androstenedione, PAL: physical activity levels

### Adjusted multivariable logistic regression on predictive factors

**Fig 2:**
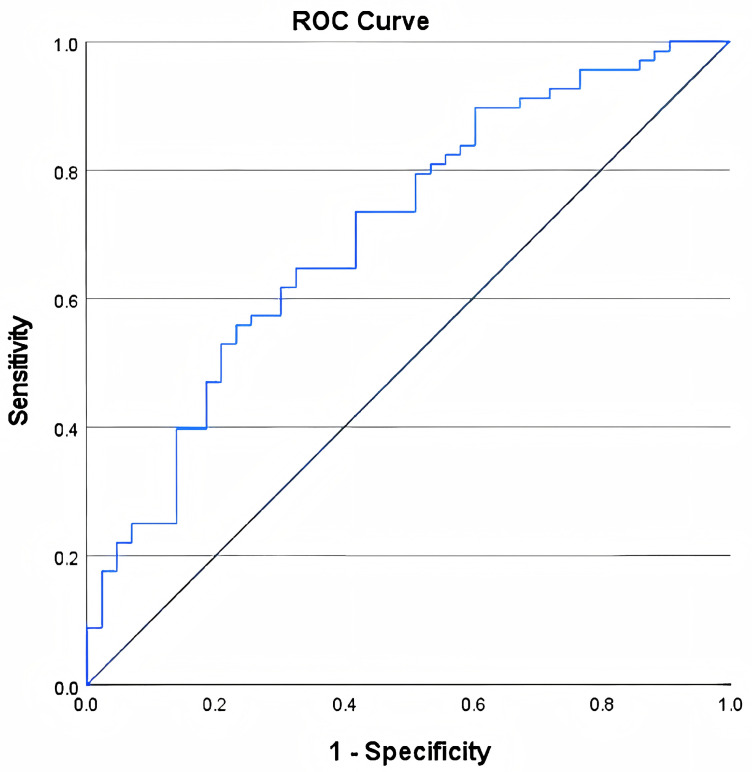
Receiver operating characteristic (ROC) curve for physical activity level (PAL) and dropout decision. Area under the curve (AUC) = 0.705 (95% CI: 0.605-0.804).

Multivariable logistic regression identified baseline PAL as the sole independent predictor of dropout after adjusting for other factors (Table 4). Each unit increase in PAL corresponded to a 98.5% lower likelihood of dropout (OR = 0.015, 95% CI: 0.001–0.352), equivalent to a 29.6% reduction per 0.3-unit increase. The model explained 19.5% of the variance (Nagelkerke R² = 0.195) and showed acceptable discrimination (AUC = 0.705, 95% CI: 0.605–0.804), with no evidence of miscalibration (Hosmer–Lemeshow p = 0.847) or multicollinearity (all VIF < 4)(Fig 2). Categorical analysis by PAL level was precluded due to the small number of participants in higher activity categories (n = 4 in dropouts, n = 5 in completers).

**Table 4:** Multivariate (adjusted) model of the determinants of dropout from lifestyle interventions.

	OR	95% CI	p-value	Tolerance	VIF
Gravidity history	4.140	0.847-20.242	0.079	0.984	1.017
BMI	1.109	0.992-1.241	0.069	0.654	1.529
PAL	0.015	0.001-0.352	0.009*	0.636	1.572

OR: odds ratio, CI: confidence interval, BMI: body mass index, PAL: physical activity levels, VIF: variance inflation factor. *: p-value<0.05.

## Discussion

This study identified baseline objectively measured PAL as the sole and strongest predictor of dropout among overweight women with PCOS undergoing lifestyle interventions. Contrary to our expectations, the network interactive platform did not improve adherence or reduce dropout rates. Notably, none of the previously reported predictors (psychological factors, androgen levels, quality of life) were replicated, underscoring the unique role of PAL in this population.

PAL is a dimensionless index reflecting total energy expenditure relative to basal metabolic rate (BMR) over 24 hours [21]. It is calculated as total energy expenditure (TEE) divided by BMR. Notably, BMR accounts for the largest component of TEE. While higher activity-induced energy expenditure(AEE) would intuitively lead to a higher PAL, overweight and obese individuals do not necessarily have lower PAL. In fact, their AEE may be similar or even higher than that of leaner individuals [22]. This apparent paradox occurs because BMR is largely determined by body size—heavier individuals expend more energy on fundamental metabolic functions (e.g., breathing, ion transport, enzyme turnover) [22].

The predictive value of PAL extends beyond PCOS. Lower objectively measured PAL is a strong predictor of all--cause mortality in chronic obstructive pulmonary disease [23], and higher PAL predicts successful long-term weight loss (2–5 years) in the general population [24,25]. However, evidence in endocrine disorders, particularly PCOS, remains limited. Of note, although we measured PAL both objectively (accelerometers) and subjectively (self-report), only the objective measure was associated with dropout. This discrepancy likely reflects common overestimation of physical activity in self-report, highlighting the need for accelerometer-based measures in clinical practice [26].

Unlike previous studies that identified psychological or biochemical predictors of dropout in women with PCOS, our findings revealed no such associations. Regarding psychological factors, our participants reported higher baseline PCOSQ scores than those in other studies using the Chi-PCOSQ [27], suggesting fewer PCOS-related negative moods and, consequently, smaller emotional differences between dropouts and completers. Regarding biochemical factors, we observed no significant differences in androgen levels between the two groups. This may be explained by ethnic variations in PCOS phenotypes: Western populations tend to present with more pronounced hyperandrogenism and hirsutism [12,13], whereas Asian women with PCOS more commonly exhibit metabolic syndrome and its related features [28]. In line with this, our participants had relatively low Ferriman Gallwey scores, further accounting for the divergence between our results and those of earlier investigations.

This study have several notable strengths. First, we assessed a comprehensive set of baseline predictors, including lifestyle characteristics often overlooked previously. This allowed us to adjust for confounders and identify objectively measured PAL, rather than baseline weight [12], as the sole dropout predictor. Second, this exploratory analysis stemmed from a randomized controlled trial, with strict inclusion criteria and standardised treatment protocols, ensured a homogeneous participant background and consistent measurements, strengthening causal inference.

A notable consideration is the high discontinuation rate observed in both the intervention and control groups, which is consistent with previous PCOS lifestyle trials [11]. Contrary to our expectations, the network interactive platform did not improve adherence or reduce dropout rates, despite a prior RCT reporting such benefits [29]. This lack of effect may be explained by habituation, whereby monthly reminders were perceived as intrusive and lost their effectiveness over time [30]. Moreover, unlike previous studies has using one-way text message reminders, our platform required mutual communication: participants had to regularly report their diet and physical activity. The time burden and stress associated with reporting unfulfilled monthly goals may have further undermined adherence. Therefore, alternative support strategies are needed. Future research should explore less intrusive reminder methods and optimal frequency thresholds to facilitate effective collaboration between providers and patients undergoing long-term lifestyle interventions.

Another consideration is the distribution of PAL categories in our sample. According to the 2004 FAO/WHO/UNU classification, PAL values of 1.40–1.69, 1.70–1.99, and 2.00–2.40 correspond to sedentary/light active, moderately active, and vigorous/very active lifestyles, respectively. Owing to the limited number of participants with higher PAL levels, we could not perform categorical analyses. Nevertheless, our continuous analysis clearly demonstrates that each 0.3-unit increase in PAL reduces dropout likelihood by approximately 30%. These findings underscores the value of objective PAL measurement over self-reported questionnaires, enabling the identification of at-risk individuals who may require additional support to improve long-term adherence.

Finally, as this is an exploratory analysis with a relatively small sample size and multiple variables examined, the p-values reported should be interpreted with caution. The hypothesis-generating nature of our study means that the observed associations, while promising, require confirmation in larger, well‑powered prospective studies. Readers are advised to view the statistical findings as supportive evidence rather than definitive conclusions.

## Conclusions

In conclusion, unlike previous studies, we identified baseline objectively measured PAL as the sole and strongest predictor of dropout in overweight women with PCOS undergoing lifestyle interventions. This finding highlights that higher PAL, assessed objectively, is associated with better treatment adherence. Clinically, we recommend routine objective screening for PAL in women with PCOS. Those with lower PAL should receive additional support alongside lifestyle interventions to improve adherence and promote weight loss.

## Supporting information

S1 File Chicklist 2025. (PDF)

S2 File Dataset. (ZIP)
