# Supplementary material for: Determinants of dropout from lifestyle interventions for overweight polycystic ovary syndrome: An exploratory analysis of randomized controlled trial
Source: PLoS One. 2026 Jun 26;21(6):e0351575. doi: 10.1371/journal.pone.0351575 (PMC13308866; doi:10.1371/journal.pone.0351575)
Supplement: Attachment — Submitted filename: Response to Reviewers.docx [file pone.0351575.s003.docx]

**Dear Editors and Reviewers:**

Thank you for your comments concerning our manuscript entitled “Determinants of Dropout from Lifestyle Interventions for Overweight Polycystic Ovary Syndrome: An Exploratory Analysis of Randomized Controlled Trial” (ID: PONE-D-25-62477). Those comments are all valuable and very helpful for revising and improving our paper, as well as the important guiding significance to our research. We have studied comments carefully and have made corrections which we hope meet with approval. According to your nice suggestions, we have made extensive corrections to our previous draft, the detailed corrections are listed below.

**Suggestions from editor：**

1. **The writing of the manuscript is not qualified for publication. Grammar modification is needed.**

Thank you for your comment regarding the writing quality of our manuscript. We have now thoroughly revised the entire manuscript for grammar and clarity. A professional English editing service was also consulted to ensure the language meets PLOS ONE’s standards. All changes are highlighted in the revised submission. We hope that the revised manuscript is now acceptable for publication.

2. **Point your main findings and contributions to the field. Clearly state your novelty.**

Thank you for the opportunity to revise our manuscript and for your valuable guidance. In response to your request to “Point your main findings and contributions to the field. Clearly state your novelty,” we have substantially revised the manuscript to highlight these aspects. Specifically:

**Main findings**: Baseline objectively measured PAL is the sole predictor of dropout; each 0.3‑unit increase reduces dropout likelihood by ~30%.

**Contributions**: First demonstration that objective PAL predicts adherence in PCOS lifestyle interventions; provides a clinical screening tool.

**Novelty**: Objectively measured PAL outperforms self‑report; identifies ethnic differences in predictors; shifts focus from weight to physical activity behaviour.

These points have been clearly articulated in the revised Discussion and in the Conclusion section. We believe that the revised manuscript now clearly communicates the main findings, contributions, and novelty of our work.

3. **If applicable, we recommend that you deposit your laboratory protocols in protocols.io to enhance the reproducibility of your results. Protocols.io assigns your protocol its own identifier (DOI) so that it can be cited independently in the future.**

Thank you for your suggestion regarding the deposit of laboratory protocols on protocols.io.

We appreciate the recommendation; however, we believe it is not applicable to our current study. As our manuscript is a secondary exploratory analysis of data from a randomized controlled trial (RCT), it did not involve the generation of new, step-by-step laboratory protocols that would be suitable for deposit on protocols.io.

**4. Please ensure that your manuscript meets PLOS ONE's style requirements, including those for file naming.**

Thank you for your reminder regarding PLOS ONE’s style requirements. We have carefully reviewed the journal’s formatting guidelines and the provided sample files. Our revised manuscript has been reformatted accordingly. We confirm that the revised submission now meets all PLOS ONE formatting requirements. Thank you for your consideration.

**5. Your ethics statement should only appear in the Methods section of your manuscript. If your ethics statement is written in any section besides the Methods, please move it to the Methods section and delete it from any other section. Please ensure that your ethics statement is included in your manuscript, as the ethics statement entered into the online submission form will not be published alongside your manuscript.**

Thank you for your careful review and for pointing out the issue regarding the ethics statement.

We have now strictly followed your suggestion and the journal’s requirement. The ethics statement has been moved entirely to the Methods section and is presented as a separate subsection titled “**Ethics statement**” immediately after “**Study design and setting**”.

We greatly appreciate your insightful feedback once again. Should the revised manuscript still fail to satisfy the journal’s criteria, we welcome you to contact us at any time.

**6. In the online submission form, you indicated that the data that support the findings of this study are available on request from the corresponding author. The data are not publicly available due to privacy of ethical restrictions.**

Thank you for your thorough review and for bringing the data availability issue to our attention. We fully understand and support PLOS ONE‘s data sharing policy.

We confirm that the minimal data set underlying the findings presented in this study has been included in the Supporting Information of the revised manuscript (S2 File. Dataset). The raw data are provided as part of the submission files, which we believe satisfies PLOS ONE’s requirement that data be “available without restriction” at the time of publication.

We have now updated the Data Availability Statement both in the online submission system and in the manuscript to accurately reflect the availability of our data.

7. **Thank you for stating the following financial disclosure:This study is supported by the Bethune Charitable Foundation and the Sichuan Key Research & Development Project (2021YFS0127) and Chengdu technological innovation research and development project (2024-YF05-00258-SN).**

**Please state what role the funders took in the study. If the funders had no role, please state: "The funders had no role in study design, data collection and analysis, decision to publish, or preparation of the manuscript."**

**If this statement is not correct you must amend it as needed.**

**Please include this amended Role of Funder statement in your cover letter; we will change the online submission form on your behalf.**

Thank you for your careful review regarding the financial disclosure statement. We have now clarified the role of the funders as requested. In our study, the funders had no role in study design, data collection and analysis, decision to publish, or preparation of the manuscript. We confirm that the required statement regarding the role of the funders has been included in our revised manuscript, as instructed.

**8.Please include captions for your Supporting Information files at the end of your manuscript, and update any in-text citations to match accordingly. Please see our Supporting Information guidelines for more information: <http://journals.plos.org/plosone/s/supporting-information.>**

At the end of the manuscript (after the References section), we have included a separate section titled “Supporting Information” with the following captions:

S2 File. Dataset.

**9. We note that there is identifying data in the Supporting Information file < IRB Approval Chinese & English.pdf, study protocol.pdf, trial study protocol Chinese & English.pdf>. Due to the inclusion of these potentially identifying data, we have removed this file from your file inventory. Prior to sharing human research participant data, authors should consult with an ethics committee to ensure data are shared in accordance with participant consent and all applicable local laws.**

**Please remove or anonymize all personal information (<specific identifying information in file to be removed>), ensure that the data shared are in accordance with participant consent, and re-upload a fully anonymized data set. Please note that spreadsheet columns with personal information must be removed and not hidden as all hidden columns will appear in the published file.**

Thank you for your note regarding identifying data in our Supporting Information files. We have removed the files IRB Approval Chinese & English.pdf, study protocol.pdf, and trial study protocol Chinese & English.pdf from our submission, as they contain personal information and are not suitable for public sharing. The only remaining Supporting Information file is a fully anonymized raw dataset (**S1 File. Chicklist 2025 and S2 File. Dataset**), from which all direct and indirect identifiers (names, dates, ID numbers, etc.) have been deleted entirely. The dataset now complies with PLOS ONE’s data policy and participant consent. We have also updated the caption accordingly. Thank you for your guidance.

10. **Additional Editor Comments :**

**As you could see, the external reviewers all suggest you revise the manuscript, right now it cannot be considered for publication. It must undergo major revision. We invite you to revise it accordingly. We kindly remind you paying special attentions on the following aspects:**

**(1) All the comments and concerns raised by all the reviewers should be considered. When you are revising, please treat all the comments and concerns seriously. DO NOT MISS or IGNORE any questions/concerns raised by the reviewers.**

**(2) A list of changes should be uploaded along with the revision (In the list, response to reviewers' as well as response to editor's comments should be included).**

Thank you for your decision and for providing the opportunity to revise our manuscript. We fully understand the need for major revision and will address all comments and concerns raised by the reviewers carefully and thoroughly. No question or concern will be missed or ignored.

We will also prepare a detailed point‑by‑point response letter (including responses to both reviewers’ and editor’s comments), as well as a marked‑up copy of the revised manuscript listing all changes. These will be uploaded together with our revised submission.

**Reviewers' comments:**

**1. Is the manuscript technically sound, and do the data support the conclusions?**

**The manuscript must describe a technically sound piece of scientific research with data that supports the conclusions. Experiments must have been conducted rigorously, with appropriate controls, replication, and sample sizes. The conclusions must be drawn appropriately based on the data presented.**

Reviewer #1: Yes

Reviewer #2: Yes

**2. Has the statistical analysis been performed appropriately and rigorously?**

Reviewer #1: Yes

Reviewer #2: Yes

**3. Have the authors made all data underlying the findings in their manuscript fully available?**

The PLOS Data policy requires authors to make all data underlying the findings described in their manuscript fully available without restriction, with rare exception (please refer to the Data Availability Statement in the manuscript PDF file). The data should be provided as part of the manuscript or its supporting information, or deposited to a public repository. For example, in addition to summary statistics, the data points behind means, medians and variance measures should be available. If there are restrictions on publicly sharing data—e.g. participant privacy or use of data from a third party—those must be specified.

Reviewer #1: Yes

Reviewer #2: Yes

**4. Is the manuscript presented in an intelligible fashion and written in standard English?**

PLOS ONE does not copyedit accepted manuscripts, so the language in submitted articles must be clear, correct, and unambiguous. Any typographical or grammatical errors should be corrected at revision, so please note any specific errors here.

Reviewer #1: Yes

Reviewer #2: Yes

**5. Review Comments to the Author**

**Reviewer #1: This was an exploratory analysis was conducted using data from a randomized controlled trial involving overweight or insulin-resistant women with PCOS aged 18–45. The investigators employed a logistic regression (univariate to multivariate) to identify dropout-associated variables. The sample size was small compared to the number of variables being investigated. However, the progression to a backward stepwise regression was perhaps the logical approach in this case. The paper was well written from the statistical perspective.**

**As a noted limitation by the authors, regrettably, the trial had a limited number of participants (n=4 and 5 in the dropout group and the group that stayed in the trial, respectively) who exhibited a moderately active, vigorous, or vigorously active lifestyle.**

**Table 4 was the final multivariable result from the exploratory approach. Also the (ROC) curve for physical activity level (PAL) and dropout decision with area under the curve (AUC) = 0.705 (95% CI: 0.605-0.804) appeared to be reasonable.. If one accepts the limitations and the exploratory approach, the study does appear to underscore the association between a higher baseline PAL and lower dropout rates for lifestyle interventions. The authors should note in the discussion the care one needs to take in interpreting the rigor of the p-values in the descriptive exploratory approach.**

**Response:** Thank you very much for your careful review and constructive comments on our manuscript. We greatly appreciate your recognition of the statistical approach and the exploratory nature of our work. Below we provide our point‑by‑point responses.

We sincerely thank you for your positive assessment of our statistical methodology. We fully agree that the sample size is relatively small. To address this limitation, we have now explicitly stated it in the Discussion section, and we have also added a cautionary note regarding the interpretation of p‑values in our exploratory analysis, as you suggested below. The backward stepwise regression was indeed chosen to reduce overfitting, and we have kept the final model parsimonious with only one independent predictor. We believe these measures help mitigate the risk of spurious findings.

Thank you for acknowledging that we had already noted the limitation regarding the limited number of participants in higher PAL categories. In the revised manuscript, we have further clarified this point in the Results section and retained a concise statement in the Discussion. Specifically, we now state that the small number of participants in higher PAL categories precluded categorical analysis, and that our findings are therefore based on continuous PAL measurements.

We are grateful for your balanced interpretation. We agree that the AUC of 0.705 indicates only acceptable discrimination, which we have now explicitly mentioned in the Results section. We have also emphasized in the Discussion that, despite the exploratory nature and limitations, our study consistently points to a meaningful association between higher baseline PAL and lower dropout rates.

Thank you for this important guidance. In response, we have added a dedicated paragraph at the end of the Discussion section, under the subheading “Interpretation of p‑values”, to explicitly caution readers about interpreting p‑values in this exploratory analysis. This addition directly addresses your concern, and we believe it appropriately reflects the rigor needed when interpreting our results.

**Reviewer #2: The manuscript aimed to identify baseline predictors of dropout in overweight women with PCOS participating in a lifestyle intervention program, and found that PAL is strongly associated with dropout decisions. It addresses an interesting topic. However, the text is somewhat verbose. Introduction, participants, baseline characteristics measurements and discussion would benefit from editing to enhance clarity and conciseness. The results presented in the table do not need to be repeated in detail in the text; a brief summary of the key findings would be sufficient. In addition, the main findings and their significance are not sufficiently highlighted. The authors are encouraged to better emphasize the key results and clearly articulate the novelty and impact of the study.**

**The details need to be carefully checked. For example, PAL has already been defined earlier in the manuscript and does not need to be written in full again at line 159.**

**Response:** Thank you very much for your insightful comments and for recognizing the interesting topic of our study. We have carefully considered all your suggestions and substantially revised the manuscript accordingly. You noted that the text was somewhat verbose, particularly in the Introduction, Participants, Baseline characteristics measurements, and Discussion sections. In response, we have thoroughly edited these sections to remove redundant content, shorten descriptions of validated questionnaires by deleting detailed reliability and validity statements, and merge repetitive sentences, thereby enhancing clarity and conciseness.

You also pointed out that the results presented in the tables do not need to be repeated in detail in the text. We have therefore revised the Results section to provide only a brief summary of key findings—for example, stating the main significant differences (PAL, BMI, acanthosis nigricans) and the key effect sizes and model performance measures, without reiterating every non-significant p-value or all variables from the tables. Regarding your comment that the main findings and their significance were not sufficiently highlighted, we have added a separate subsection in the Discussion, clearly stating that this is the first study to show that objectively measured PAL (not self-reported PAL or body weight) predicts dropout in PCOS lifestyle interventions, and we have strengthened the first paragraph of the Discussion and the Conclusion to better emphasize the novelty and clinical impact. Finally, you noted that PAL had already been defined earlier and should not be written in full again at line 159; we have carefully checked the entire manuscript and deleted that redundant full spelling, and we now use “PAL” consistently after its first definition in the Methods section. We believe these revisions have substantially improved the clarity, conciseness, and impact of our manuscript, and we thank you again for your constructive feedback.

We hereby resubmit the revised manuscript and hope that all corrections are satisfactory. Please feel free to contact us with any questions and we look forward to your decision.
