# Supplementary material for: Determinants of dropout from lifestyle interventions for overweight polycystic ovary syndrome: An exploratory analysis of randomized controlled trial
Source: PLoS One. 2026 Jun 26;21(6):e0351575. doi: 10.1371/journal.pone.0351575 (PMC13308866; doi:10.1371/journal.pone.0351575)
Supplement: Peer review history

# Peer review: Decision Letter 0

**DOI:** 10.1371/journal.pone.0351575.r001

**Authors:** Wan-Xi Yang

16 Mar 2026

PONE-D-25-62477

Determinants of Dropout from Lifestyle Interventions for Overweight Polycystic Ovary Syndrome: An Exploratory Analysis of Randomized Controlled Trial

PLOS One

Dear Dr. Zhang,

Thank you for submitting your manuscript to PLOS ONE. After careful consideration, we feel that it has merit but does not fully meet PLOS ONE’s publication criteria as it currently stands. Therefore, we invite you to submit a revised version of the manuscript that addresses the points raised during the review process.

==============================

**1. The writing of the manuscript is not qualified for publication. Grammar modification is needed.**

**2. Point your main findings and contributions to the field. Clearly state your novelty.**

==============================

Please submit your revised manuscript by Apr 30 2026 11:59PM. If you will need more time than this to complete your revisions, please reply to this message or contact the journal office at plosone@plos.org. When you’re ready to submit your revision, log on to https://www.editorialmanager.com/pone/ and select the ‘Submissions Needing Revision’ folder to locate your manuscript file.

Please include the following items when submitting your revised manuscript:

- A letter that responds to each point raised by the academic editor and reviewer(s). You should upload this letter as a separate file labeled ‘Response to Reviewers’.
- A marked-up copy of your manuscript that highlights changes made to the original version. You should upload this as a separate file labeled ‘Revised Manuscript with Track Changes’.
- An unmarked version of your revised paper without tracked changes. You should upload this as a separate file labeled ‘Manuscript’.

If you would like to make changes to your financial disclosure, please include your updated statement in your cover letter. Guidelines for resubmitting your figure files are available below the reviewer comments at the end of this letter.

If applicable, we recommend that you deposit your laboratory protocols in protocols.io to enhance the reproducibility of your results. Protocols.io assigns your protocol its own identifier (DOI) so that it can be cited independently in the future. For instructions see: https://journals.plos.org/plosone/s/submission-guidelines#loc-laboratory-protocols. Additionally, PLOS ONE offers an option for publishing peer-reviewed Lab Protocol articles, which describe protocols hosted on protocols.io. Read more information on sharing protocols at https://plos.org/protocols?utm_medium=editorial-email&utm_source=authorletters&utm_campaign=protocols.

We look forward to receiving your revised manuscript.

Kind regards,

Wan-Xi Yang, Ph.D.

Academic Editor

PLOS One

Journal Requirements:

When submitting your revision, we need you to address these additional requirements.

1. Please ensure that your manuscript meets PLOS ONE’s style requirements, including those for file naming. The PLOS ONE style templates can be found at

https://journals.plos.org/plosone/s/file?id=wjVg/PLOSOne_formatting_sample_main_body.pdf and https://journals.plos.org/plosone/s/file?id=ba62/PLOSOne_formatting_sample_title_authors_affiliations.pdf

2. Your ethics statement should only appear in the Methods section of your manuscript. If your ethics statement is written in any section besides the Methods, please move it to the Methods section and delete it from any other section. Please ensure that your ethics statement is included in your manuscript, as the ethics statement entered into the online submission form will not be published alongside your manuscript.

3. In the online submission form, you indicated that the data that support the findings of this study are available on request from the corresponding author. The data are not publicly available due to privacy of ethical restrictions.

All PLOS journals now require all data underlying the findings described in their manuscript to be freely available to other researchers, either 1. In a public repository, 2. Within the manuscript itself, or 3. Uploaded as supplementary information.

This policy applies to all data except where public deposition would breach compliance with the protocol approved by your research ethics board. If your data cannot be made publicly available for ethical or legal reasons (e.g., public availability would compromise patient privacy), please explain your reasons on resubmission and your exemption request will be escalated for approval.

4. Thank you for stating the following financial disclosure:

This study is supported by the Bethune Charitable Foundation and the Sichuan Key Research & Development Project (2021YFS0127) and Chengdu technological innovation research and development project (2024-YF05-00258-SN).

Please state what role the funders took in the study. If the funders had no role, please state: "The funders had no role in study design, data collection and analysis, decision to publish, or preparation of the manuscript."

If this statement is not correct you must amend it as needed.

Please include this amended Role of Funder statement in your cover letter; we will change the online submission form on your behalf.

5. Please include captions for your Supporting Information files at the end of your manuscript, and update any in-text citations to match accordingly. Please see our Supporting Information guidelines for more information: http://journals.plos.org/plosone/s/supporting-information.

6. We note that there is identifying data in the Supporting Information file < IRB Approval Chinese & English.pdf, study protocol.pdf, trial study protocol Chinese & English.pdf>. Due to the inclusion of these potentially identifying data, we have removed this file from your file inventory. Prior to sharing human research participant data, authors should consult with an ethics committee to ensure data are shared in accordance with participant consent and all applicable local laws.

Data sharing should never compromise participant privacy. It is therefore not appropriate to publicly share personally identifiable data on human research participants. The following are examples of data that should not be shared:

-Name, initials, physical address

-Ages more specific than whole numbers

-Internet protocol (IP) address

-Specific dates (birth dates, death dates, examination dates, etc.)

-Contact information such as phone number or email address

-Location data

-ID numbers that seem specific (long numbers, include initials, titled “Hospital ID”) rather than random (small numbers in numerical order)

Data that are not directly identifying may also be inappropriate to share, as in combination they can become identifying. For example, data collected from a small group of participants, vulnerable populations, or private groups should not be shared if they involve indirect identifiers (such as sex, ethnicity, location, etc.) that may risk the identification of study participants.

Additional guidance on preparing raw data for publication can be found in our Data Policy (https://journals.plos.org/plosone/s/data-availability#loc-human-research-participant-data-and-other-sensitive-data) and in the following article: http://www.bmj.com/content/340/bmj.c181.long.

Please remove or anonymize all personal information (<specific identifying information in file to be removed>), ensure that the data shared are in accordance with participant consent, and re-upload a fully anonymized data set. Please note that spreadsheet columns with personal information must be removed and not hidden as all hidden columns will appear in the published file.

7. If the reviewer comments include a recommendation to cite specific previously published works, please review and evaluate these publications to determine whether they are relevant and should be cited. There is no requirement to cite these works unless the editor has indicated otherwise.

Additional Editor Comments :

As you could see, the external reviewers all suggest you revise the manuscript, right now it cannot be considered for publication. It must undergo major revision. We invite you to revise it accordingly. We kindly remind you paying special attentions on the following aspects:

(1) All the comments and concerns raised by all the reviewers should be considered. When you are revising, please treat all the comments and concerns seriously. DO NOT MISS or IGNORE any questions/concerns raised by the reviewers.

(2) A list of changes should be uploaded along with the revision (In the list, response to reviewers’ as well as response to editor’s comments should be included).

[Note: HTML markup is below. Please do not edit.]

Reviewers’ comments:

Reviewer’s Responses to Questions

**Comments to the Author**

1. Is the manuscript technically sound, and do the data support the conclusions?

The manuscript must describe a technically sound piece of scientific research with data that supports the conclusions. Experiments must have been conducted rigorously, with appropriate controls, replication, and sample sizes. The conclusions must be drawn appropriately based on the data presented.

Reviewer #1: Yes

Reviewer #2: Yes

**********

2. Has the statistical analysis been performed appropriately and rigorously?

Reviewer #1: Yes

Reviewer #2: Yes

**********

3. Have the authors made all data underlying the findings in their manuscript fully available?

The PLOS Data policy requires authors to make all data underlying the findings described in their manuscript fully available without restriction, with rare exception (please refer to the Data Availability Statement in the manuscript PDF file). The data should be provided as part of the manuscript or its supporting information, or deposited to a public repository. For example, in addition to summary statistics, the data points behind means, medians and variance measures should be available. If there are restrictions on publicly sharing data—e.g. participant privacy or use of data from a third party—those must be specified.

Reviewer #1: Yes

Reviewer #2: Yes

**********

4. Is the manuscript presented in an intelligible fashion and written in standard English?

PLOS ONE does not copyedit accepted manuscripts, so the language in submitted articles must be clear, correct, and unambiguous. Any typographical or grammatical errors should be corrected at revision, so please note any specific errors here.

Reviewer #1: Yes

Reviewer #2: Yes

**********

5. Review Comments to the Author

Please use the space provided to explain your answers to the questions above. You may also include additional comments for the author, including concerns about dual publication, research ethics, or publication ethics. (Please upload your review as an attachment if it exceeds 20,000 characters)

Reviewer #1: This was an exploratory analysis was conducted using data from a randomized controlled trial involving overweight or insulin-resistant women with PCOS aged 18–45. The investigators employed a logistic regression (univariate to multivariate) to identify dropout-associated variables. The sample size was small compared to the number of variables being investigated. However, the progression to a backward stepwise regression was perhaps the logical approach in this case. The paper was well written from the statistical perspective.

As a noted limitation by the authors, regrettably, the trial had a limited number of participants (n=4 and 5 in the dropout group and the group that stayed in the trial, respectively) who exhibited a moderately active, vigorous, or vigorously active lifestyle.

Table 4 was the final multivariable result from the exploratory approach. Also the (ROC) curve for physical activity level (PAL) and dropout decision with area under the curve (AUC) = 0.705 (95% CI: 0.605-0.804) appeared to be reasonable.. If one accepts the limitations and the exploratory approach, the study does appear to underscore the association between a higher baseline PAL and lower dropout rates for lifestyle interventions. The authors should note in the discussion the care one needs to take in interpreting the rigor of the p-values in the descriptive exploratory approach.

Reviewer #2: The manuscript aimed to identify baseline predictors of dropout in overweight women with PCOS participating in a lifestyle intervention program, and found that PAL is strongly associated with dropout decisions. It addresses an interesting topic. However, the text is somewhat verbose. Introduction, participants, baseline characteristics measurements and discussion would benefit from editing to enhance clarity and conciseness. The results presented in the table do not need to be repeated in detail in the text; a brief summary of the key findings would be sufficient. In addition, the main findings and their significance are not sufficiently highlighted. The authors are encouraged to better emphasize the key results and clearly articulate the novelty and impact of the study.

The details need to be carefully checked. For example, PAL has already been defined earlier in the manuscript and does not need to be written in full again at line 159.

**********

6. PLOS authors have the option to publish the peer review history of their article (what does this mean?). If published, this will include your full peer review and any attached files.

If you choose “no”, your identity will remain anonymous but your review may still be made public.

**Do you want your identity to be public for this peer review?**  For information about this choice, including consent withdrawal, please see our Privacy Policy.

Reviewer #1: No

Reviewer #2: No

**********

[NOTE: If reviewer comments were submitted as an attachment file, they will be attached to this email and accessible via the submission site. Please log into your account, locate the manuscript record, and check for the action link "View Attachments". If this link does not appear, there are no attachment files.]

To ensure your figures meet our technical requirements, please review our figure guidelines: https://journals.plos.org/plosone/s/figures

You may also use PLOS’s free figure tool, NAAS, to help you prepare publication quality figures: https://journals.plos.org/plosone/s/figures#loc-tools-for-figure-preparation.

NAAS will assess whether your figures meet our technical requirements by comparing each figure against our figure specifications.

# Author comment: Author response to Decision Letter 1

**DOI:** 10.1371/journal.pone.0351575.r002

20 Apr 2026

Dear Reviewers:

Thank you for your comments concerning our manuscript entitled “Determinants of Dropout from Lifestyle Interventions for Overweight Polycystic Ovary Syndrome: An Exploratory Analysis of Randomized Controlled Trial” (ID: PONE-D-25-62477). Those comments are all valuable and very helpful for revising and improving our paper, as well as the important guiding significance to our research. We have studied comments carefully and have made corrections which we hope meet with approval. According to your nice suggestions, we have made extensive corrections to our previous draft, the detailed corrections are listed below.

Reviewer #1: This was an exploratory analysis was conducted using data from a randomized controlled trial involving overweight or insulin-resistant women with PCOS aged 18–45. The investigators employed a logistic regression (univariate to multivariate) to identify dropout-associated variables. The sample size was small compared to the number of variables being investigated. However, the progression to a backward stepwise regression was perhaps the logical approach in this case. The paper was well written from the statistical perspective.

As a noted limitation by the authors, regrettably, the trial had a limited number of participants (n=4 and 5 in the dropout group and the group that stayed in the trial, respectively) who exhibited a moderately active, vigorous, or vigorously active lifestyle.

Table 4 was the final multivariable result from the exploratory approach. Also the (ROC) curve for physical activity level (PAL) and dropout decision with area under the curve (AUC) = 0.705 (95% CI: 0.605-0.804) appeared to be reasonable.. If one accepts the limitations and the exploratory approach, the study does appear to underscore the association between a higher baseline PAL and lower dropout rates for lifestyle interventions. The authors should note in the discussion the care one needs to take in interpreting the rigor of the p-values in the descriptive exploratory approach.

Response: Thank you very much for your careful review and constructive comments on our manuscript. We greatly appreciate your recognition of the statistical approach and the exploratory nature of our work. Below we provide our point‑by‑point responses.

We sincerely thank you for your positive assessment of our statistical methodology. We fully agree that the sample size is relatively small. To address this limitation, we have now explicitly stated it in the Discussion section, and we have also added a cautionary note regarding the interpretation of p‑values in our exploratory analysis, as you suggested below. The backward stepwise regression was indeed chosen to reduce overfitting, and we have kept the final model parsimonious with only one independent predictor. We believe these measures help mitigate the risk of spurious findings.

Thank you for acknowledging that we had already noted the limitation regarding the limited number of participants in higher PAL categories. In the revised manuscript, we have further clarified this point in the Results section and retained a concise statement in the Discussion. Specifically, we now state that the small number of participants in higher PAL categories precluded categorical analysis, and that our findings are therefore based on continuous PAL measurements.

We are grateful for your balanced interpretation. We agree that the AUC of 0.705 indicates only acceptable discrimination, which we have now explicitly mentioned in the Results section. We have also emphasized in the Discussion that, despite the exploratory nature and limitations, our study consistently points to a meaningful association between higher baseline PAL and lower dropout rates.

Thank you for this important guidance. In response, we have added a dedicated paragraph at the end of the Discussion section, under the subheading “Interpretation of p‑values”, to explicitly caution readers about interpreting p‑values in this exploratory analysis. This addition directly addresses your concern, and we believe it appropriately reflects the rigor needed when interpreting our results.

Reviewer #2: The manuscript aimed to identify baseline predictors of dropout in overweight women with PCOS participating in a lifestyle intervention program, and found that PAL is strongly associated with dropout decisions. It addresses an interesting topic. However, the text is somewhat verbose. Introduction, participants, baseline characteristics measurements and discussion would benefit from editing to enhance clarity and conciseness. The results presented in the table do not need to be repeated in detail in the text; a brief summary of the key findings would be sufficient. In addition, the main findings and their significance are not sufficiently highlighted. The authors are encouraged to better emphasize the key results and clearly articulate the novelty and impact of the study.

The details need to be carefully checked. For example, PAL has already been defined earlier in the manuscript and does not need to be written in full again at line 159.

Response: Thank you very much for your insightful comments and for recognizing the interesting topic of our study. We have carefully considered all your suggestions and substantially revised the manuscript accordingly. You noted that the text was somewhat verbose, particularly in the Introduction, Participants, Baseline characteristics measurements, and Discussion sections. In response, we have thoroughly edited these sections to remove redundant content, shorten descriptions of validated questionnaires by deleting detailed reliability and validity statements, and merge repetitive sentences, thereby enhancing clarity and conciseness.

You also pointed out that the results presented in the tables do not need to be repeated in detail in the text. We have therefore revised the Results section to provide only a brief summary of key findings—for example, stating the main significant differences (PAL, BMI, acanthosis nigricans) and the key effect sizes and model performance measures, without reiterating every non-significant p-value or all variables from the tables. Regarding your comment that the main findings and their significance were not sufficiently highlighted, we have added a separate subsection in the Discussion, clearly stating that this is the first study to show that objectively measured PAL (not self-reported PAL or body weight) predicts dropout in PCOS lifestyle interventions, and we have strengthened the first paragraph of the Discussion and the Conclusion to better emphasize the novelty and clinical impact. Finally, you noted that PAL had already been defined earlier and should not be written in full again at line 159; we have carefully checked the entire manuscript and deleted that redundant full spelling, and we now use “PAL” consistently after its first definition in the Methods section. We believe these revisions have substantially improved the clarity, conciseness, and impact of our manuscript, and we thank you again for your constructive feedback.

We hereby resubmit the revised manuscript and hope that all corrections are satisfactory. Please feel free to contact us with any questions and we look forward to your decision.

Attachment Submitted filename: Response to Reviewers.docx

# Peer review: Decision Letter 1

**DOI:** 10.1371/journal.pone.0351575.r003

**Authors:** Wan-Xi Yang

28 May 2026

Determinants of Dropout from Lifestyle Interventions for Overweight Polycystic Ovary Syndrome: An Exploratory Analysis of Randomized Controlled Trial

PONE-D-25-62477R1

Dear Dr. Zhang,

We’re pleased to inform you that your manuscript has been judged scientifically suitable for publication and will be formally accepted for publication once it meets all outstanding technical requirements.

Within one week, you’ll receive an e-mail detailing the required amendments. When these have been addressed, you’ll receive a formal acceptance letter and your manuscript will be scheduled for publication.

An invoice will be generated when your article is formally accepted. Please note, if your institution has a publishing partnership with PLOS and your article meets the relevant criteria, all or part of your publication costs will be covered. Please make sure your user information is up-to-date by logging into Editorial Manager at Editorial Manager® and clicking the ‘Update My Information’ link at the top of the page. For questions related to billing, please contact billing support.

If your institution or institutions have a press office, please notify them about your upcoming paper to help maximize its impact. If they’ll be preparing press materials, please inform our press team as soon as possible -- no later than 48 hours after receiving the formal acceptance. Your manuscript will remain under strict press embargo until 2 pm Eastern Time on the date of publication. For more information, please contact onepress@plos.org.

Kind regards,

Wan-Xi Yang, Ph.D.

Academic Editor

PLOS One

Additional Editor Comments (optional):

Reviewers’ comments:

Reviewer’s Responses to Questions

**Comments to the Author**

1. If the authors have adequately addressed your comments raised in a previous round of review and you feel that this manuscript is now acceptable for publication, you may indicate that here to bypass the “Comments to the Author” section, enter your conflict of interest statement in the “Confidential to Editor” section, and submit your “Accept” recommendation.

Reviewer #1: All comments have been addressed

Reviewer #2: All comments have been addressed

**********

2. Is the manuscript technically sound, and do the data support the conclusions?

The manuscript must describe a technically sound piece of scientific research with data that supports the conclusions. Experiments must have been conducted rigorously, with appropriate controls, replication, and sample sizes. The conclusions must be drawn appropriately based on the data presented.

Reviewer #1: (No Response)

Reviewer #2: Yes

**********

3. Has the statistical analysis been performed appropriately and rigorously?

Reviewer #1: (No Response)

Reviewer #2: I Don’t Know

**********

4. Have the authors made all data underlying the findings in their manuscript fully available?

The PLOS Data policy requires authors to make all data underlying the findings described in their manuscript fully available without restriction, with rare exception (please refer to the Data Availability Statement in the manuscript PDF file). The data should be provided as part of the manuscript or its supporting information, or deposited to a public repository. For example, in addition to summary statistics, the data points behind means, medians and variance measures should be available. If there are restrictions on publicly sharing data—e.g. participant privacy or use of data from a third party—those must be specified.

Reviewer #1: (No Response)

Reviewer #2: Yes

**********

5. Is the manuscript presented in an intelligible fashion and written in standard English?

PLOS ONE does not copyedit accepted manuscripts, so the language in submitted articles must be clear, correct, and unambiguous. Any typographical or grammatical errors should be corrected at revision, so please note any specific errors here.

Reviewer #1: (No Response)

Reviewer #2: Yes

**********

6. Review Comments to the Author

Please use the space provided to explain your answers to the questions above. You may also include additional comments for the author, including concerns about dual publication, research ethics, or publication ethics. (Please upload your review as an attachment if it exceeds 20,000 characters)

Reviewer #1: (No Response)

Reviewer #2: (No Response)

**********

7. PLOS authors have the option to publish the peer review history of their article (what does this mean?). If published, this will include your full peer review and any attached files.

If you choose “no”, your identity will remain anonymous but your review may still be made public.

**Do you want your identity to be public for this peer review?**  For information about this choice, including consent withdrawal, please see our Privacy Policy.

Reviewer #1: No

Reviewer #2: No

**********

# Editor report: Acceptance letter

**DOI:** 10.1371/journal.pone.0351575.r004

**Authors:** Wan-Xi Yang

PONE-D-25-62477R1

PLOS One

Dear Dr. Zhang,

I’m pleased to inform you that your manuscript has been deemed suitable for publication in PLOS One. Congratulations! Your manuscript is now being handed over to our production team.

At this stage, our production department will prepare your paper for publication. This includes ensuring the following:

* All references, tables, and figures are properly cited

* All relevant supporting information is included in the manuscript submission,

* There are no issues that prevent the paper from being properly typeset

You will receive further instructions from the production team, including instructions on how to review your proof when it is ready. Please keep in mind that we are working through a large volume of accepted articles, so please give us a few days to review your paper and let you know the next and final steps.

Lastly, if your institution or institutions have a press office, please let them know about your upcoming paper now to help maximize its impact. If they’ll be preparing press materials, please inform our press team within the next 48 hours. Your manuscript will remain under strict press embargo until 2 pm Eastern Time on the date of publication. For more information, please contact onepress@plos.org.

You will receive an invoice from PLOS for your publication fee after your manuscript has reached the completed accept phase. If you receive an email requesting payment before acceptance or for any other service, this may be a phishing scheme. Learn how to identify phishing emails and protect your accounts at https://explore.plos.org/phishing.

If we can help with anything else, please email us at customercare@plos.org.

Thank you for submitting your work to PLOS ONE and supporting open access.

Kind regards,

PLOS ONE Editorial Office Staff

on behalf of

Dr. Wan-Xi Yang

Academic Editor

PLOS One
